# Perforated Blind Pouch: An Unusual Late Complication Following Lateral Anastomosis After a Right Hemicolectomy

**DOI:** 10.7759/cureus.15044

**Published:** 2021-05-15

**Authors:** Bishal Pal, Souradeep Dutta, Ankit Jain, Abhinaya Reddy, Vishnu Prasad Nelamangala Ramakrishnaiah

**Affiliations:** 1 Surgery, Jawaharlal Institute of Postgraduate Medical Education and Research, Puducherry, IND

**Keywords:** blind loop syndrome, blind pouch syndrome, blind pouch, micro-perforation, bacterial peritonitis, gastrointestinal perforation

## Abstract

Blind loop syndrome (BLS) is a well-recognized delayed complication in small bowel strictures, stenosis, fistulas, diverticula, or post-gastrectomy afferent loop syndrome. However, due to its delayed presentation, BLS after side-to-side bowel anastomosis is inadequately reported. The vicious cycle of the blind loop is due to bacterial overgrowth, resulting in diarrhea, weight loss, malnutrition, and rarely mucosal erosion, bleeding, and perforation peritonitis. Diagnosis of BLS requires knowledge of previous surgery performed, a high level of clinical suspicion, and experienced radiological abilities. In this case report, we present the clinico-radiological profile of a 54-year-old diabetic patient with a perforated blind ileal pouch occurring four years after a right hemicolectomy with side-to-side ileo-transverse anastomosis.

## Introduction

“Blind loop syndrome” (BLS) and related complications due to intestinal overgrowth and abnormal peristalsis in the blind pouch are well recognized. BLS has been more commonly reported in small bowel strictures, stenosis, fistulas, diverticula, or after post-gastrectomy afferent loop syndrome [1,2]. However, due to its delayed presentation, BLS is rarely reported following a side-to-side intestinal anastomosis. Thus, its actual incidence after the side-to-side intestinal anastomosis is unknown. Here, we present the clinico-radiological features of a diabetic patient presenting with blind pouch perforation following a side-to-side ileo-transverse anastomosis.

## Case presentation

A 54-year-old diabetic gentleman presented with severe diffuse abdominal pain with abdominal distension for four days, followed by diarrhea and fever with chills for two days. He had undergone a right hemicolectomy for carcinoma cecum in a different hospital four years back. Postoperative biopsy reported well-differentiated adenocarcinoma with pathological staging - T4N2M0. He received eight cycles of adjuvant capecitabine and oxaliplatin, following which he was under six-monthly follow-up with no recurrence.

**Figure 1:**
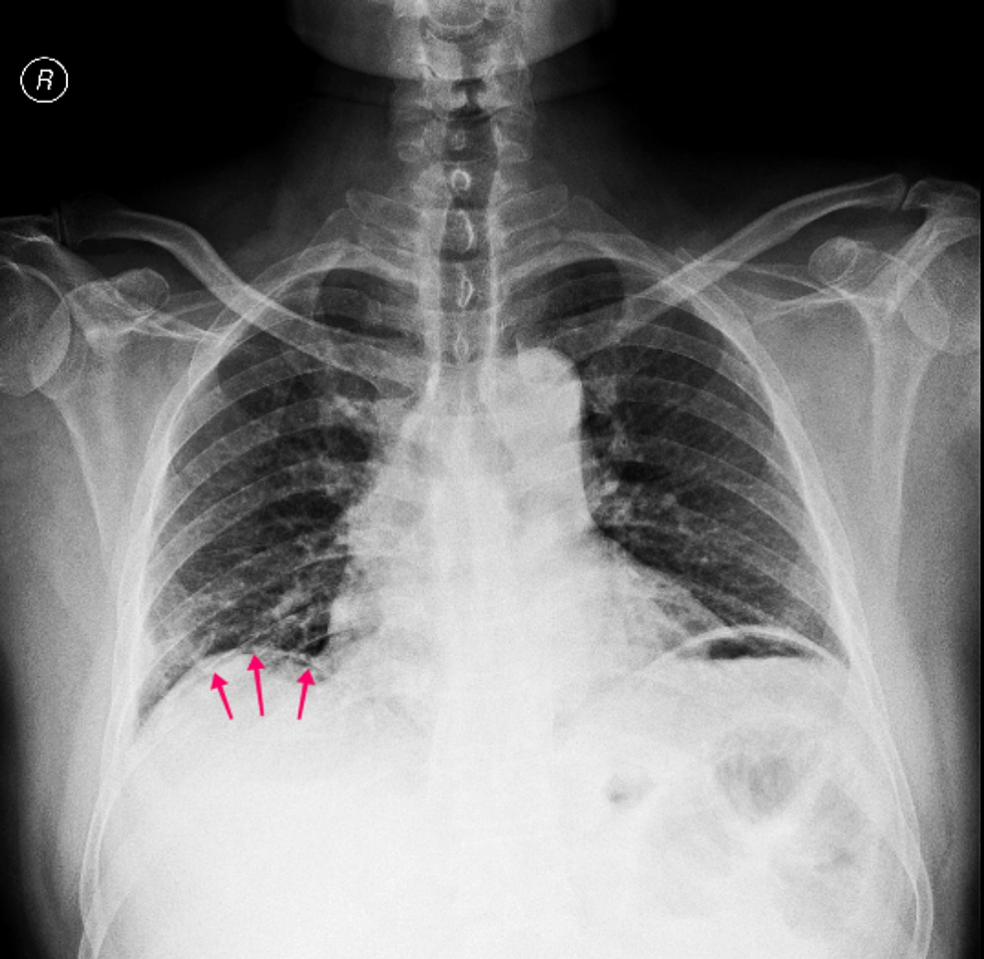
Chest X-ray showing a thin rim of air under the right diaphragm (red arrows).

On examination, he was conscious, oriented, and dehydrated. He had tachycardia (110/min) with a blood pressure of 130/70 mm of Hg. The abdomen was distended, with diffuse tenderness and signs of peritonitis. His blood glucose at the time of presentation was 550 mg/dl with no ketoacidosis. Blood investigations showed hemoglobin of 13 gm/dl, total counts of 7600/mm^3^ with 81% neutrophils, platelet counts were 2.2 lacs/mm^3^, urea of 41 mg/dl, creatinine of 0.67 mg/dl, sodium of 138 mEq/dl, and potassium of 3.58 mEq/dl. An erect chest X-ray showed a thin rim of pneumoperitoneum under the right hemidiaphragm (Figure 1).

**Figure 2:**
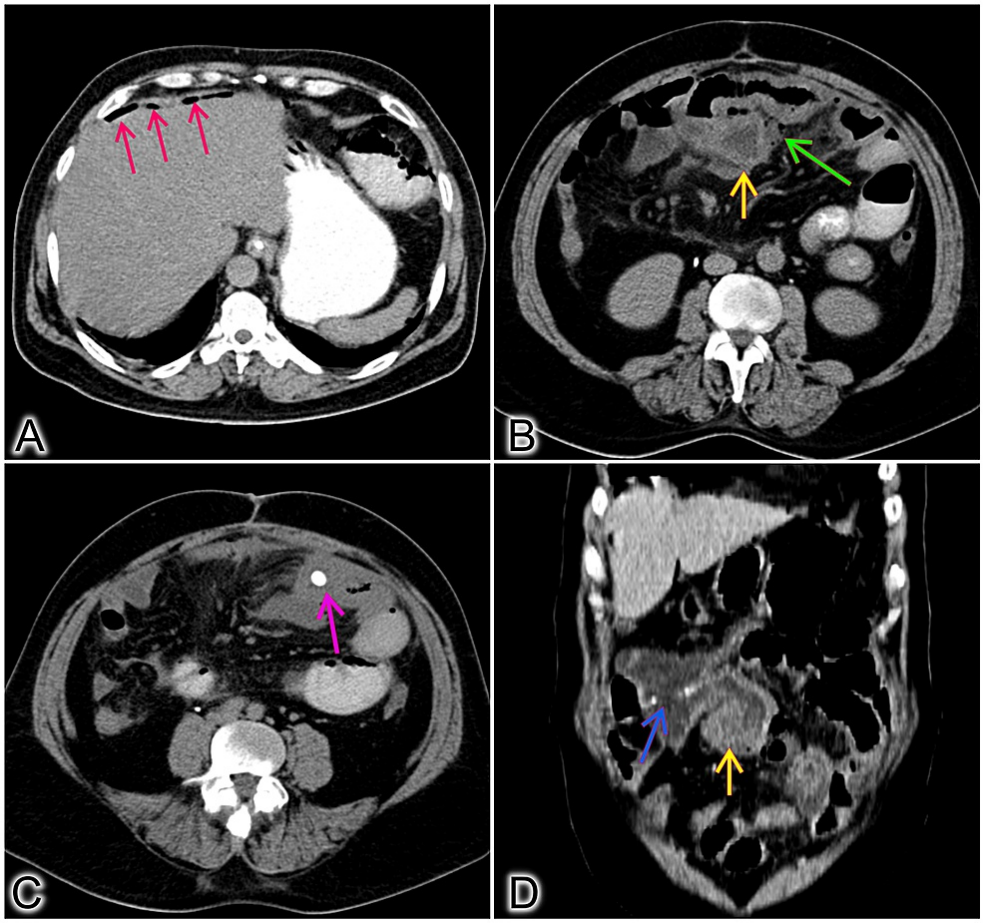
CT images. (A) Red arrows showing specs of intra-abdominal air. (B) Yellow arrow shows the thickened inflamed blind ileal loop, green arrow shows extra-luminal air with collection adjacent to the blind loop. (C) Enterolith seen in the colonic blind loop portion. (D) Coronal section showing the thickened ileal loop (yellow arrow) and the ileo-transverse lateral anastomosis (blue arrow).

A contrast-enhanced computed tomography (CECT) was done, which showed scattered foci of pneumoperitoneum, and edematous thickened ileal loop distal to the ileo-transverse anastomosis with a small collection with air foci within it, mesenteric fat stranding but no free fluid abdomen. The anastomotic site was healthy with no abnormal thickening suggestive of recurrence (Figure 2).

**Figure 3:**
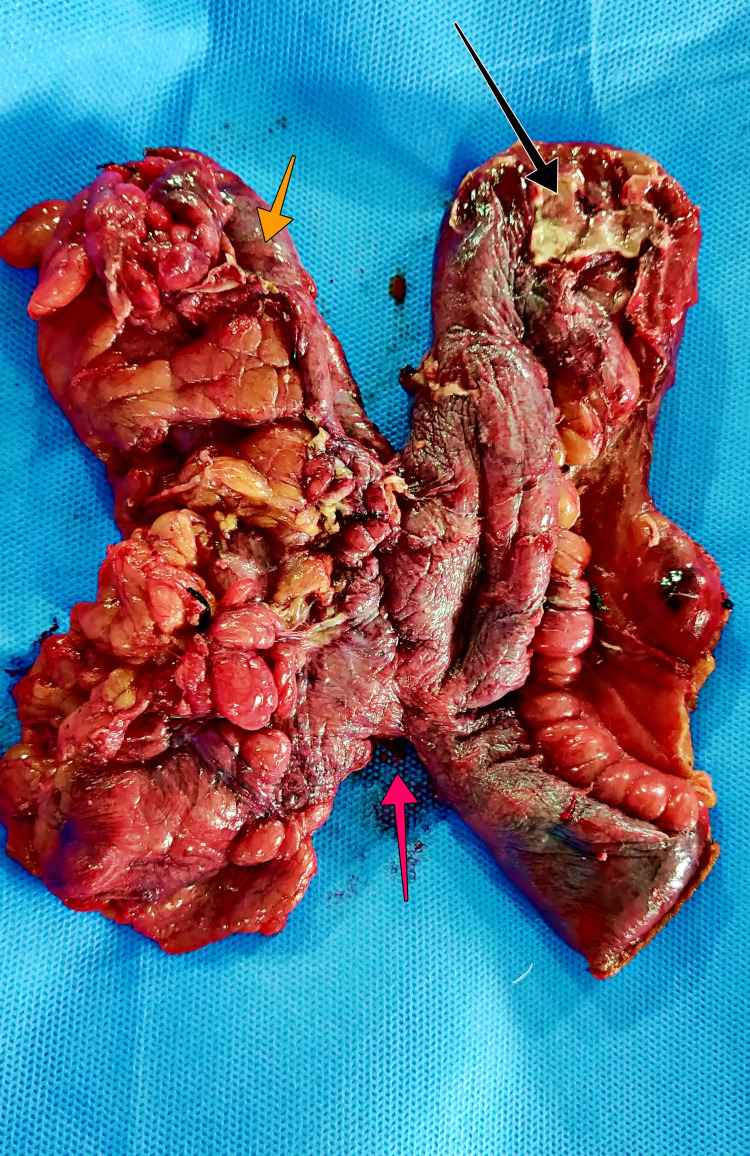
Resected specimen. Black arrow – Dilated ileal blind loop with pus flakes and perforation. Yellow arrow – Colonic blind loop. Red arrow – Side-side anastomosis.

After the initial resuscitation, he was taken up for an exploratory laparotomy. Intraoperatively, an unhealthy, thickened, distended ileal blind loop of length 8 cm with a sloughed-off portion of the bowel wall covered with pus flakes with a pinpoint micro-perforation, distal to the side-to-side ileo-transverse colon anastomosis (Figure 3).

Resection of the previous anastomotic site and a redo functional end-to-end (anatomical side-to-side) ileo-transverse anastomosis was done using a linear stapler. The postoperative course was uneventful. The patient was discharged on a regular diet on the sixth postoperative day.

## Discussion

Bowel continuity, post intestinal resection, can be restored in different anatomical configurations by various surgical techniques. End-to-end anastomosis is the most physiological but has complications like increase risk of anastomotic leak due to devascularization of ends of the bowel during mesenteric dissection and discrepancies in bowel circumference like during ileocolic anastomosis [3]. There has been an increase in side-to-side lateral anastomosis owing to the popularity of linear staplers. Though it is an easy, fast, accurate, and reliable technique of re-establishing intestinal continuity, it is not without complications [3]. An excess segment of the bowel left distal to the anastomosis starts the vicious cycle of a blind pouch. Circular muscle fibers that get divided during such a side-to-side anastomosis can result in local dysmotility with stasis of enteric content and enterolith formation. Subsequent progressive distension causing thinning of the blind pouch’s wall coupled with bacterial overgrowth causing inflammation and mucosal ulceration may lead to bleeding or bowel perforation [4]. However, not all blind pouches are symptomatic. There are reports of incidental long blind loops discovered during autopsy studies or surgical interventions [5]. Comorbidities like diabetes [6], amyloidosis [7], scleroderma, hypothyroidism [8] disrupt intestinal motility and therefore promote bacterial overgrowth, thus adding to the effect of the anatomical anomaly [9]. In the current case, the patient had diabetes.

After an extensive literature search, 14 cases of pinpoint perforation of blind pouches were found, presenting with signs and symptoms of peritonitis [5,10-12]. BLS has a delayed presentation after the side-to-side anastomosis. According to a review by Frank et al. published in 1990, most patients with symptomatic blind pouch presented more than five years after the surgery [11]. In another series of three patients, patients presented with blind loop perforation around two years after the surgery [12].

Diagnosis of BLS is challenging and requires a high level of clinical suspicion. Patients can present with chronic diarrhea, steatorrhea, vitamin B12 deficiency causing megaloblastic anemia, and other nutritional deficiencies [13]. A patient with a history of side-to-side intestinal anastomosis, presenting with spontaneous onset of acute abdominal pain and diarrhea with or without signs of peritonitis, should be suspected with BLS. An X-ray may or may not show pneumoperitoneum. Therefore, such patients should undergo further cross-sectional imaging. On CECT, a blind pouch is seen as a saccular enteric structure with thickened walls, with surgical staples seen nearby [14]. In cases with blind pouch perforation, there might be evidence of enteroliths and surrounding collection around the cul-de-sac with extra-luminal air foci [12].

The possibility of creating a blind pouch should be kept in mind while doing a side-to-side anastomosis. The length of end loops distal to anastomosis should be kept minimum, preferably closing the loops just distal to the lateral anastomosis. In symptomatic cases, excision of the diseased segment and re-anastomosis, preferably in an end-to-end fashion, should be performed [15]. Limited resection of the diseased pouch can also be attempted without the need for a resection anastomosis depending on the condition of the diseased segment [12]. An end-to-end anastomosis, despite its limitations, is free from the complication of BLS [3]. In a retrospective analysis of ileocolic anastomosis, nine distended blind loops were identified in 31 side-to-side anastomoses, whereas no blind loops were observed after 12 end-to-end anastomoses [16].

## Conclusions

In the era of stapled intestinal anastomosis, the possibility of a BLS occurring after a side-to-side anastomosis should not be neglected. Though this is a well-established long-term complication, traditional surgical textbooks have not given much importance to it till now. For prevention of BLS, the length of end loops distal to anastomosis should be kept minimum. In symptomatic cases, excision of the diseased segment and re-anastomosis, preferably in an end-to-end fashion, should be performed.
